# RADIATE – Radial Dysplasia Assessment, Treatment and Aetiology: protocol for the development of a core outcome set using a Delphi survey

**DOI:** 10.1186/s13063-019-3459-4

**Published:** 2019-06-10

**Authors:** George R. F. Murphy, Malcolm Logan, Gill Smith, Bran Sivakumar

**Affiliations:** 1 Department of Plastic Surgery, Great Ormond Street Hospital for Children, London, WC1N 3JH UK; 2 Randall Division of Cell and Molecular Biophysics, Guy’s Campus, King’s College London, London, SE1 1UL UK

**Keywords:** Radial dysplasia, Congenital upper limb anomalies, core outcome set, Congenital hand surgery

## Abstract

**Background:**

Radial dysplasia (RD) is a disfiguring, potentially disabling congenital upper limb anomaly. Multiple surgical techniques are in current use, with little agreement on the optimal treatment approach. At present, no core outcome set exists specifically for RD, and the literature is dominated by retrospective case series. A recent systematic review by this group demonstrated significant heterogeneity on which outcomes are measured and how they are measured.

**Methods/design:**

The RADIATE study will conduct a three-round online Delphi process, involving adult RD patients, the parents of children with RD, hand surgeons and hand therapists. The initial list of outcomes was drawn from our recent systematic review and will be supplemented by suggestions from the stakeholder groups. Following the Delphi process, outcomes that meet the consensus in definition will be ratified at a final consensus meeting. We will then follow the COSMIN guidelines to select outcome measurement instruments. Where appropriate, these will overlap with the outcome measures specified in the forthcoming standard set for congenital upper limb anomalies published by the International Consortium for Health Outcomes Measurement.

**Discussion:**

The Radial Dysplasia Assessment, Treatment and Aetiology (RADIATE) study aims to address the uncertainty in the treatment of RD, and to begin to answer the question ‘What is the most appropriate treatment of the forearm and hand for children with RD?’ by establishing a core outcome set.

**Trial registration:**

COMET initiative study, 902. Registered in May 2016.

**Electronic supplementary material:**

The online version of this article (10.1186/s13063-019-3459-4) contains supplementary material, which is available to authorized users.

## Background

Radial dysplasia (RD) is a disfiguring, potentially disabling congenital upper limb anomaly, affecting approximately 1 in 8000 births [1–3]. It is characterised by the variable absence or hypoplasia of the pre-axial upper limb skeleton (radius and thumb) and soft tissues [4]. Affected children have a phenotype ranging from isolated thumb hypoplasia to complete absence of the thumb and radius, with severe ulnar bowing, elbow stiffness and humeral hypoplasia. Children may be unilaterally or bilaterally affected. Known causes include spontaneous mutations, teratogenic drugs and syndromes such as Holt–Oram syndrome, vertebral, anal, cardiac, tracheo-esophageal, renal and limb (VACTERL) anomalies or Fanconi anaemia, although approximately 50% of cases are of unknown aetiology. Children without associated major comorbidities can expect a normal lifespan.

Globally, several treatment techniques to address this wrist deformity are in common use at specialist centres, including centralisation [5] or radialisation [6] of the ulna, either with or without prior soft-tissue distraction [7], and the microvascular transfer of a toe joint to act as a radial buttress to the wrist [8]. The surgical treatment of the soft tissues is highly variable. A recent systematic review by our group found that patients suffer poor forearm growth and some degree of recurrent radial wrist deviation, whether treated surgically or conservatively [9]. Currently, there is no core outcome set (COS) specifically for RD, although a generalised standard set for congenital upper limb anomalies is due to be published soon [10]. Measuring outcomes is further complicated because the limbs change during growth, necessitating follow-ups until skeletal maturity before the final outcome can be assessed.

The Radial Dysplasia Assessment, Treatment and Aetiology (RADIATE) study aims to address the uncertainty in the treatment of RD, and to begin to answer the question ‘What is the most appropriate treatment of the forearm and hand for children with RD?’ by establishing a COS.

### Selection of outcomes for use in clinical studies of RD

Clinical studies, whether interventional or observational, should have prospectively defined primary and secondary outcomes that answer the questions posed by the hypothesis. However, for existing studies of RD, the outcomes measured are numerous and highly variable between studies. The techniques for measuring many outcomes are also poorly defined, making it difficult to compare studies or synthesise their results in a meta-analysis. It is also unclear how relevant, if at all, these outcomes are to patients.

### Outcome reporting bias

Another problem, especially in the surgical literature, is outcome reporting bias [11], where a large number of outcomes are measured but only those that show interesting or positive results are reported. This presents a biased view of the results of a trial. As the number of tests increases, so does the risk of results arising by chance erroneously being labelled as significant.

### Core outcome sets

The development of a COS is one way to overcome these problems. Such a prospectively defined group of outcomes is the minimum dataset that trials for a given condition should report. For paediatric conditions, they are ideally developed with patient and family involvement. Prospectively specifying the outcomes and how they are measured should prevent cherry-picking of positive results and it should standardise studies, allowing comparison and synthesis of their results. When developed with patients and their families, they also provide reassurance that the outcomes are relevant to patients.

### Scope, aim and objectives

#### Aim

The aim of this study is to develop an initial COS suitable for assessing treatment outcomes after any form of treatment, including conservative management, for congenital upper limb anomalies in patients with RD.

#### Scope

The COS is designed for use in both research and routine clinical care, in any health-care system treating congenital upper limb anomalies due to RD. It should cover children of all ages and adults, and should apply to all interventions for congenital upper limb anomalies in patients with RD.

#### Objectives

The specific study objectives are: (1) to list all outcomes previously reported in studies of the treatment of RD, identified through a systematic review of the literature, (2) to prioritise outcomes from the overall perspective of patients, parents and clinicians, (3) to compare outcomes that are important to patients and parents with those that are important to clinicians and (4) to integrate these outcomes into a combined COS.

## Methods/design

### Systematic review

We recently published a systematic review of the long-term outcomes of both surgical and conservative treatments for RD [9]. This was prospectively registered with the PROSPERO database (CRD42016036665) and conducted using the Cochrane highly sensitive search strategy. We searched Medline and Embase via OvidSP, PubMed, Cochrane, ClinicalTrials.gov and the WHO International Clinical Trials portal for published and unpublished studies. Searches were not restricted by date or language. From all studies identified that reported outcomes for RD treatment, we extracted a list of outcomes measured for RD following the process in the COMET handbook [12] (Section 2.7.1.2), which will form the starting point for our Delphi process.

### Identification of outcomes important to patients, parents and clinicians

#### Overview

To achieve consensus on a COS for RD within and between groups, we propose to use an online Delphi process, adapted and simplified from the protocol laid out by Harman et al. [13]. This will include four groups of participants:

- RD patients aged over 16
- parents of RD patients aged under 16
- hand therapists who treat RD patients
- hand surgeons who treat RD patients

**Fig. 1:**
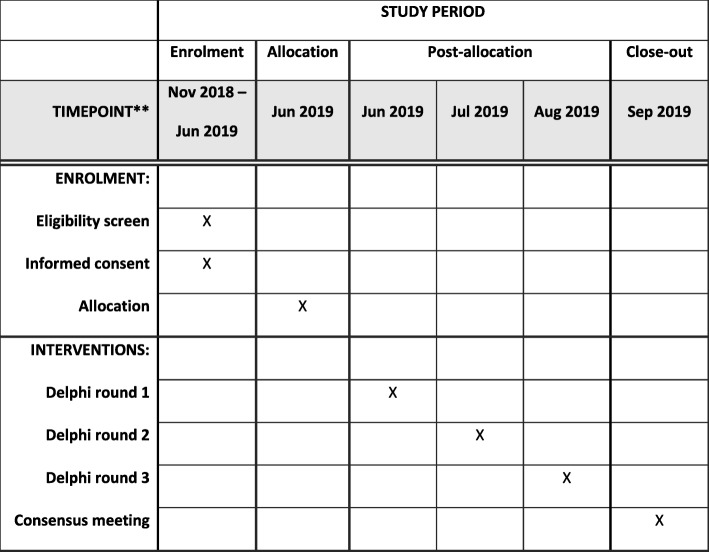
SPIRIT figure; schedule of enrolment, interventions and assessments

These groups were chosen so that patient and family perspectives will have the same status as surgical and therapist perspectives. We aim to make the groups of similar size. Patient and parent groups will be drawn from across the UK, and clinician groups drawn from specialist centres internationally. The study will be managed from Great Ormond St Hospital in London, and participants will be recruited via email by the central research team, following identification by participating specialist centres worldwide. The Delphi process will be administered using the secure software DelphiManager at the University of Liverpool. The study process is summarised in Fig. 1 and Additional file 1.

### Identification of potential outcomes

Our initial outcome list was generated from the outcomes identified during our systematic review. We included all outcomes that we identified in the global literature on RD treatment. Composite outcome scores have been split into their component parts, where possible. The outcome list will be presented thematically, by outcome domain. Outcomes will be described using lay language, then medical language beneath, with a diagram or picture as required. The draft outcome list will be piloted with members of all stakeholder groups before use to ensure it is easily understood and clear.

### Participants and stakeholder selection

We aim to recruit the patient and parent groups via specialist centres across the UK, and the therapist and surgeon groups from global specialist centres. All participants will be required to be proficient with spoken and written English, and to have access to a computer and internet connection.

### Delphi process

#### Round 1: initial ranking and finalising outcomes

Participant identification centres have been identified by the review authors. Potential participants will be identified by each centre locally, then invited to participate in the Delphi process by the central research team at Great Ormond St. Those who agree will be invited to register with DelphiManager, then sent an email linking to an online survey. Each identified outcome will be listed thematically by outcome domain. An open question at the end will allow participants to nominate important missing outcomes. They will be asked to rank each outcome 1–9, where 1–3 are ‘not important’, 4–6 are ‘important but not critical’ and 7–9 are ‘critical’. A review author (GM), who will not participate in the survey, can see who has completed the online survey. Participants will be given 3 weeks to complete the survey. Reminder emails will be sent after 1 and after 2 weeks.

#### Analysis of round 1

Newly suggested outcomes will be reviewed by two review authors (GM and BS) to ensure they are genuinely novel, then combined and added to the round 2 questionnaire accordingly. The results will be analysed by participant group and as an overall summary, which will include the number participating and the distribution of scores per outcome. Outcomes meeting the consensus out definition will be excluded from the round 2 questionnaire. Individual participation in round 2 will be contingent upon completing the survey in round 1.

#### Round 2

Participants will again be contacted by email with a link to the online survey. For each previously scored outcome, they will be presented with a histogram summary of the responses for each group and for all groups combined, plus a reminder of their previous score. They will then be asked to re-score each outcome, again from 1 to 9, and then to score any newly suggested outcomes identified in round 1. Participants will be given 3 weeks to complete the round 2 survey, with a reminder email being sent after 1 and after 2 weeks.

#### Analysis of round 2

The results will be analysed both by participant group and as an overall summary, which will include the number participating and the distribution of scores per outcome. Outcomes meeting the consensus out definition will be excluded from the round 3 questionnaire. Individual participation in round 3 will again be contingent upon completing the survey in round 2.

#### Round 3

Participants will again be contacted by email with a link to the online survey. For each outcome they will be presented with a histogram summary of the responses for each group and for all groups combined, plus a reminder of their round 2 score. They will then be asked to re-score each outcome, again from 1 to 9. Participants will be given 3 weeks to complete the round 3 survey, with a reminder email being sent after 1 and after 2 weeks.

#### Analysis of round 3

The results will be analysed both by participant group and as an overall summary, which will include the number participating and the distribution of scores per outcome. Outcomes will be classified as consensus in, consensus out or no consensus using the criteria from Harman et al. [13], as summarised in Table 1. The distribution of scores and consensus result for each outcome will be displayed by group and overall and used to structure the final consensus meeting.

**Table 1:** Definition of consensus (after Harman et al. [13])

Consensus classification	Description	Definition
Consensus in	Consensus that the outcome should be included in the core outcome set	70% or more of the participants score it as 7–9, and <15% of the participants score it as 1–3
Consensus out	Consensus that the outcome should not be included in the core outcome set	70% or more of the participants score it as 1–3, and <15% of the participants score it as 7–9
No consensus	Uncertainty about the importance of the outcome	Any other outcome

#### Consensus meeting

A final consensus will be reached during a consensus meeting, which may involve a mixture of face-to-face and teleconference participation. All participants in the Delphi survey will be invited. All participants will receive the results of round 3 in advance, presented by group and overall. We will then follow the COSMIN guidelines to select outcome measurement instruments. To avoid duplication of effort by patients, clinicians and researchers, where outcomes overlap, we will use measurement instruments from the International Consortium for Health Outcomes Measurement (ICHOM) standard set for congenital upper limb anomalies. The final COS will be published in a peer-reviewed journal.

### Statistical analysis and sample size

Scores for each item will be presented as a histogram of responses, and the percentage of responses in each group (1–3, 4–6 and 7–9) calculated. This will be done by individual stakeholder group and for all groups combined. As there is no standard model for the sample size required for a Delphi process, we will aim for between 10 and 15 participants per group, with the patient and parent groups covering patients with varying degrees of disease severity and different ages.

## Discussion

There is currently no COS specifically for RD. This study seeks to develop one, with the involvement of a wide range of participants, to ensure maximal acceptability to both patients and clinicians.

As with any project, this study has limitations. The long list of outcomes has been developed from a comprehensive systematic review, but it is possible that we have missed relevant outcomes, especially those not represented in the existing literature. Supplementing the initial list with qualitative interviews is one approach suggested in the COMET handbook [12], but is beyond the resources of this project. By allowing all stakeholders to suggest further outcomes in round 1 of the Delphi process, we aim to reduce this risk to acceptable levels, but must concede that this approach “does not have the same standing as the knowledge generated by [qualitative] research” [12].

The choice of stakeholders has been informed by current treatment protocols for RD. Primary surgery typically happens before the patient is aged 2, meaning that parents are the key early decision makers, whose views we wish to include. We aim to reflect the independent views of RD children both by encouraging parents to involve their children as much as is age and developmentally appropriate in completing the survey, and by including a separate group of RD patients aged over 16. Together with the international groups of surgeons and hand therapists, we believe this provides a balanced perspective on RD treatment.

Where outcomes overlap, we will use measurement instruments from the recently developed ICHOM congenital upper limb anomalies standard set [10], so that patients and clinicians are not unduly burdened by multiple measurements for an outcome. This standard set includes measurement instruments that were chosen through a methodologically robust Delphi process, informed by patient focus groups and the literature.

We hope that this outcome set will make the interpretation, comparison and synthesis of future studies easier.

### Study status

The study protocol is version 1.0 (18 July 2018). Recruitment commenced on 28 November 2018 and is expected to be complete before June 2019. The study is expected to take 3 months once recruitment is complete.

## Additional file

Additional file 1: SPIRIT 2013 Checklist: Recommended items to address in a clinical trial protocol and related documents*. (DOC 121 kb)

## Data Availability

The datasets produced, used and analysed during the current study will be available in anonymised form at individual participant level from the corresponding author on reasonable request, after publication of the final study results.

## Abbreviations

COS: Core outcome set
ICHOM: International Consortium for Health Outcomes Measurement
RADIATE: Radial Dysplasia: Assessment, Treatment and Aetiology
RD: Radial dysplasia
VACTERL: Vertebral, anal, cardiac, tracheo-esophageal, renal and limb anomalies
